# Plain x‐ray misses many ureteric calculi: Time to challenge the old dogma?

**DOI:** 10.1002/bco2.192

**Published:** 2022-09-26

**Authors:** Stephen McGeorge, Brendon Loh, Ryan Shibu, Karen Dobeli, Nathan J. Brown, Rachel Esler, Craig Hacking, Megan Purvey, Matthew J. Roberts

**Affiliations:** ^1^ Department of Urology Royal Brisbane and Women's Hospital Brisbane Queensland Australia; ^2^ Faculty of Medicine University of Queensland Brisbane Queensland Australia; ^3^ Emergency and Trauma Centre Royal Brisbane and Women's Hospital Brisbane Queensland Australia; ^4^ Department of Medical Imaging Royal Brisbane and Women's Hospital Brisbane Queensland Australia; ^5^ Faculty of Medicine University of Queensland Centre for Clinical Research Brisbane Queensland Australia

**Keywords:** calculi, CTKUB, emergency department, length of stay, ureteric, urolithiasis, x‐ray

## 1 INTRODUCTION

Ureteric colic is a common emergency department (ED) presentation and usually diagnosed with noncontrast computed tomography (CT). Plain abdominal x‐ray (AXR) was historically used for diagnosis and may help to monitor position of radio‐opaque calculi during conservative management in contemporary practice. Despite being fast, convenient and having low ionising radiation exposure compared with CT, the clinical usefulness of AXR in contemporary practice is unclear. Investigations with little clinical value may unnecessarily impede patient flow and increase ED wait‐times and length of stay (LOS). The aim of this study was to determine the clinical benefits of AXR in ED patients with ureteric calculi for follow‐up purposes and the impacts of AXR on ED LOS.

Information about patients with ureteric colic who presented to the Royal Brisbane and Women's Hospital ED between October 2019 and September 2020 was retrieved from the Emergency Department Information System and matched with information from radiology departmental archives for patients who underwent CT and AXR. Ureteric colic presentations were identified by diagnosis codes for ‘renal colic’ and ‘urinary calculus’. Patients who did not have ureteric calculi on CT were excluded. The visibility of ureteric calculi on AXR was determined by radiologist interpretation and review by the authors. Demographic, radiological and logistical data were collected. Univariate statistical analysis was performed. Institutional ethics exemption was granted (EX/2021/QRBW/79710).

During the 12‐month study period, 252 ED patients (202 male) were diagnosed with ureteric calculi on CT (mean size: 4.8 mm; 95%CI 4.5–5.2 mm) with 160 (63.5%) located in the distal ureter. The median (interquartile range; IQR) age of patients was 46 (33–56) years.

A total of 107 patients (42.4%) had AXR in addition to CT, with most AXR (93.5%, *n =* 100/107) performed after CT per our institutional outpatient follow‐up protocol for ureteric calculi. People who received AXR tended to be younger (median age 39 vs. 48 years, *p <* 0.01) with smaller calculi (mean 4.2 mm, 95%CI 3.9–4.5 vs. 5.4 mm, 95%CI 4.9–5.9; *p <* 0.01) than those who underwent CT alone. Calculi in those who had AXR were located most commonly in the distal ureter (than proximal ureter; 44.1% vs. 26.2%).

**FIGURE 1:**
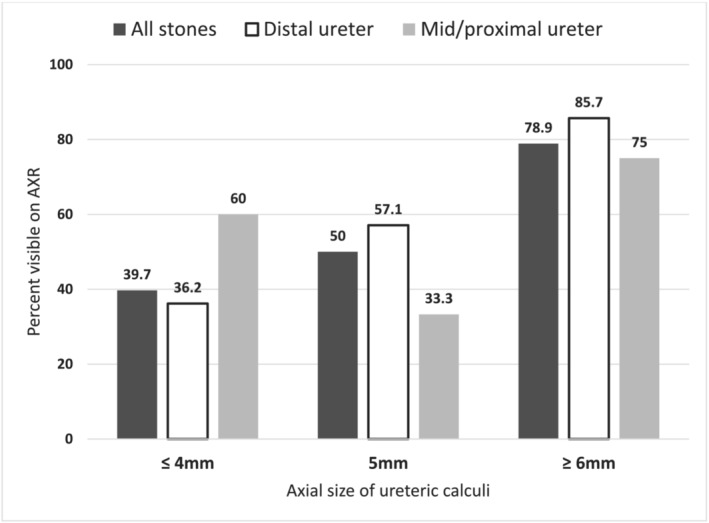
Percentage of ureteric calculi visible on abdominal x‐ray (AXR) in 107 emergency department patients investigated for ureteric colic, according to axial diameter and location

Over half of ureteric calculi detected with CT were not identified on AXR (48.6% identified, *n =* 52/107) despite CT images being available to the AXR reporting radiologist. Review of AXR by the authors identified fewer calculi (41.1%, *n =* 44/107) with disagreement for 12 AXR, of which 10 was the inability to identify calculi, so the greater figure was used to represent the best‐case scenario. Calculi visible on AXR tended to be larger (mean size 4.61 vs. 3.76 mm, *p =* 0.003) and located in the proximal ureter (60.7%, *n =* 17/28) rather than mid or distal ureter (44.3%, *n =* 35/79). Smaller calculi were identified less frequently (Figure 1), with 39.7% (*n =* 27/68) of calculi ≤4 mm identified on AXR, which was lower again for distal ureteric calculi at 36.2% (*n =* 21/58).

The mean ED LOS was longer for patients who underwent both AXR and CT (521 min, 95%CI 458–584) than those who had CT alone (417 min, 95%CI 380–455; *p <* 0.01) despite no difference in time from arrival to CT being performed (mean 171 min, 95%CI 154–189 vs. 163 min, 95%CI 148–179, *p =* 0.5). Patients who had AXR were also more likely to be admitted to the ED short stay unit than patients with CT alone (65.4% vs. 31.7%, *p <* 0.01), where similar increases in LOS were observed (453 min, 95%CI 375–530 vs. 347 min, 95%CI 288–406; *p =* 0.03).

Discharge to home occurred in 95.3% of patients with AXR and CT, compared with 60.7% for patients with CT alone. This would be expected given the smaller size and higher proportion of distal calculi in those who had AXR and CT. Over a quarter of patients with both AXR and CT (*n =* 30, 28%) were discharged within 30 min of AXR being performed, implying this was a final requirement before discharge as part of the local follow‐up protocol.

The low yield of AXR should challenge its place in imaging pathways, especially given the impact on LOS. Surveillance is the typical indication for AXR in ureteric colic. Given the high discharge rate for patients with AXR and CT, it appears that AXR was predominantly for follow‐up purposes according to the institutional protocol requiring AXR before discharge. Small calculi in the distal ureter are more likely to be suitable for conservative management and therefore have AXR performed as part of a follow‐up protocol. However, these same factors also mean that these calculi are less likely to be visible on AXR, with only 36.2% of distal ureteric calculi ≤4 mm being identified on AXR. There was comparatively greater yield for AXR in identifying proximal calculi of any size (60.7%, *n =* 17/28, *p =* 0.04) and any calculi that were 6 mm or larger (78.9%, *n =* 15/19, *p <* 0.01) compared with distal calculi ≤4 mm. As such, AXR has limited utility for follow‐up of small distal ureteric calculi but may be considered in larger or proximal ureteric calculi.

Detection of calculi on AXR also depends on radio‐opacity due to calcium content. Analysis of calculi from 2009 to 2011 in Australia found that 16% of upper tract calculi are uric acid composition, which would be radiolucent, and this has remained largely static since the 1970s.^1^ In a study of patients with a history of calcium‐containing calculi requiring urological intervention, 75% of calculi were detectable with AXR, although there was a higher calculi size (median 7 mm, IQR 5‐10 mm) than our results and there were at least two reviewers of each AXR.^2^ As such, there may be more of a role for AXR in patients with an established history of recurrent calcium‐based calculi. There may also be local resource constraints mandating use of AXR, such as in remote areas. One study reported that AXR can be useful in deciding between endoscopic management or extracorporeal shockwave lithotripsy using fluoroscopy guidance, although the availability of lithotripters with ultrasound guidance may reduce this utility.^3^, ^4^

While AXR has lower ionising radiation exposure than standard dose CT, ultralow dose CT (ULD CT) achieves comparable exposure while reliably identifying ureteric calculi, including smaller distal ureteric calculi. Ultralow dose CT has a mean effective dose of 1.02–1.04 mSv in contemporary series (<1.9 mSv by definition) while maintaining diagnostic accuracy of 95.5%.^5^, ^6^, ^7^ The effective dose of AXR can be 0.5–1 mSv, and one retrospective series reported that 34% of AXR exposed patients to higher effective doses than their concurrent CT.^6^, ^8^ As such, a pathway incorporating ULD CT where available, in place of AXR, for ureteric calculi follow‐up purposes may result in similar levels of ionising radiation exposure. Recurrent stone formers may also have multiple CT scans, so this must still be used judiciously.

In conclusion, we found that the use of AXR in ureteric colic increased total ED LOS, and rates and lengths of admission to the ED short stay unit yet failed to identify many ureteric calculi even with CT imaging available to readers. The expected advantages of AXR in speed and relatively low ionising radiation exposure are counteracted by low accuracy and increased ED LOS. Ultralow dose CT is a more accurate alternative to AXR while maintaining low effective doses. In the context of increased demand on EDs and heavier hospital workloads, improvement in patient flow by optimising imaging pathways for ureteric calculi should be given close consideration.

## CONFLICT OF INTEREST

There are no actual or potential conflicts of interest in relation to this article.
